# Effects of Taiji Stick exercise on strength, balance, and activities of daily living in older adults: a randomized controlled trial

**DOI:** 10.3389/fpubh.2025.1647055

**Published:** 2025-09-23

**Authors:** Longfei Cao, Xiaoxiao Dong, Kai Qi, Chunhui Zhou, Aiguo Chen

**Affiliations:** ^1^Gdansk University of Physical Education and Sport, Gdansk, Poland,; ^2^Jiangsu Vocational Institute of Commerce, Nanjing, China; ^3^Nanjing Sport Institute, Nanjing, China; ^4^Shanghai University of Sport, Shanghai, China

**Keywords:** Taiji Stick, older adults, grip strength, lower extremity strength, dynamic balance ability, activities of daily living

## Abstract

**Background:**

With advancing age, older adults experience an accelerated decline in physical function. The progressive decline not only compromises overall well-being but also precipitates numerous serious complications, including an increased likelihood of falls and bone injuries, and in more advanced stages, a diminished ability to carry out daily tasks independently. Numerous studies have shown that engagement in Health Qigong and Taijiquan exercises contributes to better physical functioning and facilitates the completion of everyday activities in senior populations. The positive outcomes observed in previous studies provide the theoretical foundation and practical basis for the current investigation. An 11-week Taiji Stick exercise regimen was implemented to explore its impact on grip and lower limb strength, dynamic balance, and daily functional activities in older individuals.

**Methods:**

A randomized controlled design was employed. Thirty-five older adults were randomly assigned to either an intervention group or a control group. The intervention group engaged in an 11-week Taiji Stick exercise program, held three times weekly for 45 min per session. Participants in the control group continued with their routine daily activities without receiving any form of intervention. Grip strength was assessed using a portable digital dynamometer. Lower limb muscular strength was evaluated through the Five Times Sit-to-Stand test (5STS), while dynamic balance was measured via the Timed Up and Go test (TUG). The Barthel Index served to examine participant’s activities of daily living (ADL).

**Results:**

(1) After the intervention, the experimental group showed no significant change in right-hand grip strength [pre-test: 19.00 (15.65, 24.45), post-test: 19.40 (16.15, 25.20); *p* = 0.185, |*r*| ≈ 0.32], whereas the control group exhibited a significant decrease in right-hand grip strength [pre-test: 17.15 (14.18, 23.68), post-test: 16.75 (13.03, 23.48); *p* = 0.001, |*r*| ≈ 0.79]. (2) Following the intervention, the experimental group demonstrated significant improvements in lower limb strength [pre-test: 15.00 (13.40, 20.00), post-test:12.30 (9.55, 15.20); *p* < 0.001, |*r*| ≈ 0.86], dynamic balance (pre-test: 15.13 ± 4.04, post-test: 11.77 ± 3.42; *p* < 0.001, η_p_^2^ = 0.618), as well as in daily living capacity [pre-test: 95.00 (90.00, 100.00), post-test: 95.00 (92.50, 100.00); *p* < 0.05, |*r*| ≈ 1.00]. In comparison, the control group experienced a marked decline in lower limb strength [pre-test:16.40 (14.75, 20.85), post-test:17.50 (15.50, 23.25); *p* < 0.001, |*r*| ≈ 0.88], as well as a notable deterioration in activities of daily living [pre-test: 95.00 (88.75, 100.00), post-test: 90.00 (80.00, 95.00); *p* < 0.01, |*r*| ≈ 0.89], along with a non-significant reduction in dynamic balance was noted (pre-test:17.16 ± 3.92, post-test:17.92 ± 4.70; *p* > 0.05, η_p_^2^ = 0.081).

**Conclusion:**

An 11-week Taiji Stick exercise program can effectively enhance lower limb strength and dynamic balance, maintain upper limb strength, show potential to reduce fall-related risks, and improve daily living ability in older adults.

**Clinical trial registration:**

This study was approved for registration by the Chinese Clinical Trial Registry on April 24, 2024 (ChiCTR2400083424).

## 1 Introduction

The WHO predicts that by 2050, the worldwide number of people 65 years of age and older will reach 1.6 billion (1, 2). Between 2000 and 2030, the proportion of individuals 65 years of age and older in the United States is expected to rise from 12.4 to 19.6%, while in Europe, it will increase from 15.5 to 24.3% (3). By the close of 2024, the number of people 65 years of age and older in China surpassed 220 million, constituting 15.6% of the national population (4). These statistics highlight the increasingly severe trend of global population aging.

Aging leads to progressive skeletal muscle atrophy and frailty (5), which in turn contribute to declines in lower limb strength, impaired balance, and gait disturbances—primary causes often linked to falls in older adult individuals (6, 7). Around 30% of community-dwelling individuals 65 years of age and older suffer a fall each year (8), while nearly 50% of those aged 80 and above fall more than once annually (9). Falls in the older adult not only cause injuries, disability, and reduced quality of life, but also impose a heavy financial burden on healthcare systems. For instance, in 2019, fall-related incidents among older adults in the Netherlands incurred medical expenses exceeding one billion euros (10). Therefore, enhancing lower limb strength, balance, and gait stability is essential for preventing falls, improving physical function, and promoting independence in daily activities among the older adult.

Key strategies aimed at enhancing or postponing the deterioration of physical abilities in the older adult population include pharmacological treatments, hormone replacement, nutritional supplements, and exercise interventions (11–13). Among the above interventions, exercise is notable for being affordable, widely accessible, and having few to no side effects, making it an increasingly favored approach among the older adult population. Currently, exercise interventions aimed at improving postural stability and leg muscle strength among the older adult primarily include perturbation training, dual- or multi-task balance drill, progressive resistance exercise, Tai Chi, and Qigong exercises (14, 15). Among these, perturbation-based balance training involves the intentional application of external disturbances, such as treadmill-based exercises, mechanical pulling, or manual push and pull forces, to improve an individual’s reactive balance control in response to instability (16, 17). Dual-task balance training combines a balance exercise with either a cognitive or motor task, whereas multi-task balance training integrates both cognitive and motor tasks simultaneously (14, 18). Existing studies have demonstrated that perturbation-based balance training and dual−/multi-task balance training, as forms of exercise intervention, can effectively improve one or more intrinsic factors linked to the likelihood of falling in older adults, particularly lower limb strength, balance capacity, and gait stability. However, some studies have indicated that among community-dwelling older adult individuals, perturbation-based balance training does not demonstrate significantly greater effectiveness than general care (physiotherapy) in enhancing stability control or alleviating fear of falls (10), and its impact on actual fall incidence and other fall-related indicators appears to be limited (19). Additionally, research has shown that although 12 weeks multi-task balance drill can improve response speed in healthy older adults, it does not lead to significant improvements in postural sway (20). Another study found that 6-week multi-task balance training enhanced working memory in healthy older adults, though it did not significantly improve balance performance (21). On the other hand, systematic reviews have also indicated that while progressive resistance training (i.e., strength training involving gradually increasing loads) can significantly enhance muscle strength in older adults, its effectiveness in improving balance remains uncertain or subject to debate (22, 23).

In summary, although each of the aforementioned exercise interventions has its own advantages, Tai Chi and Health Qigong are more suitable for older adults in terms of comfort during exercise and ease of long-term adherence, due to their gentle and moderate movements, low risk, and minimal reliance on specific venues or equipment.

Taiji Stick, along with Tai Chi, Baduanjin, and Wuqinxi, all belong to the domain of Chinese traditional sports. Among these, Taiji Stick, Baduanjin, and Wuqinxi have been officially recognized and promoted by the General Administration of Sport of China as standardized Health Qigong. It is worth emphasizing that Taiji Stick is the only routine among the 11 officially endorsed Health Qigong sets that involves practice with a handheld stick (24). The Taiji Stick shares a common origin and similar movement patterns with Tai Chi, Baduanjin, and Wuqinxi, providing both the theoretical rationale and practical foundation for this study. Numerous studies have demonstrated that mind–body exercises with similar movement mechanics improve functional ability; thus, we hypothesized that Taiji Stick exercise would lead to statistically significant improvements in lower limb strength and dynamic balance ability compared to controls in senior care center-dwelling older adults after 11 weeks. In order to examine this hypothesis, the present research employed a randomized controlled trial design for the first time, using the Taiji stick as an exercise intervention to assess their effects on strength, balance, and daily activity function in the aging population. A gap in the existing literature is being addressed by this study regarding the impact of apparatus-based Health Qigong interventions on physical function in the older adult and holds both exploratory and pioneering significance.

## 2 Methods

### 2.1 Study design and ethical approval

A mixed factorial design (2 × 2) was employed, with Group (Experimental vs. Control) as the between-subjects factor and Time (Pre- vs. Post-intervention) as the within-subjects factor. The experimental procedure consisted of three phases: baseline assessment, an 11-week Taiji Stick exercise intervention, and post-intervention outcome assessment. Ethical approval for this study was obtained from the Human Research Ethics Committee of Nanjing Sport Institute (Approval No. RT-2025-04).

### 2.2 Participants

The sample size was estimated using G*Power 3.1 software for a repeated-measures ANOVA (Es = 0.25, α = 0.05, Power = 0.80), the calculation indicated that a minimum of 34 older adults is needed. In this study, the expected effect size was set at 0.25, which falls within the range of moderate effect size. This setting not only aligns with the practical needs of effect detection in intervention studies but also takes full account of the general effect size levels in similar clinical researches (25). Researchers recruited older adults following predefined inclusion and exclusion criteria. The inclusion criteria consisted of: (1) individuals 65 years of age or older, regardless of gender. (2) physically intact limbs. (3) educational level of junior high school or above. (4) no prior experience with Taiji stick exercise. (5) participation in the study was voluntary with signed informed consent. The criteria for exclusion included: (1) the existence of severe physical or mental disorders. (2) substantial visual impairment. (3) inability to stand or walk independently without assistive devices. (4) A history of lower extremity fracture within the preceding 6 months. Participants subsequently underwent exclusion from the final analysis if they withdrew from the study before completion, or were absent for three consecutive sessions or accumulated a total of six absences during the intervention period.

**Figure 1:**
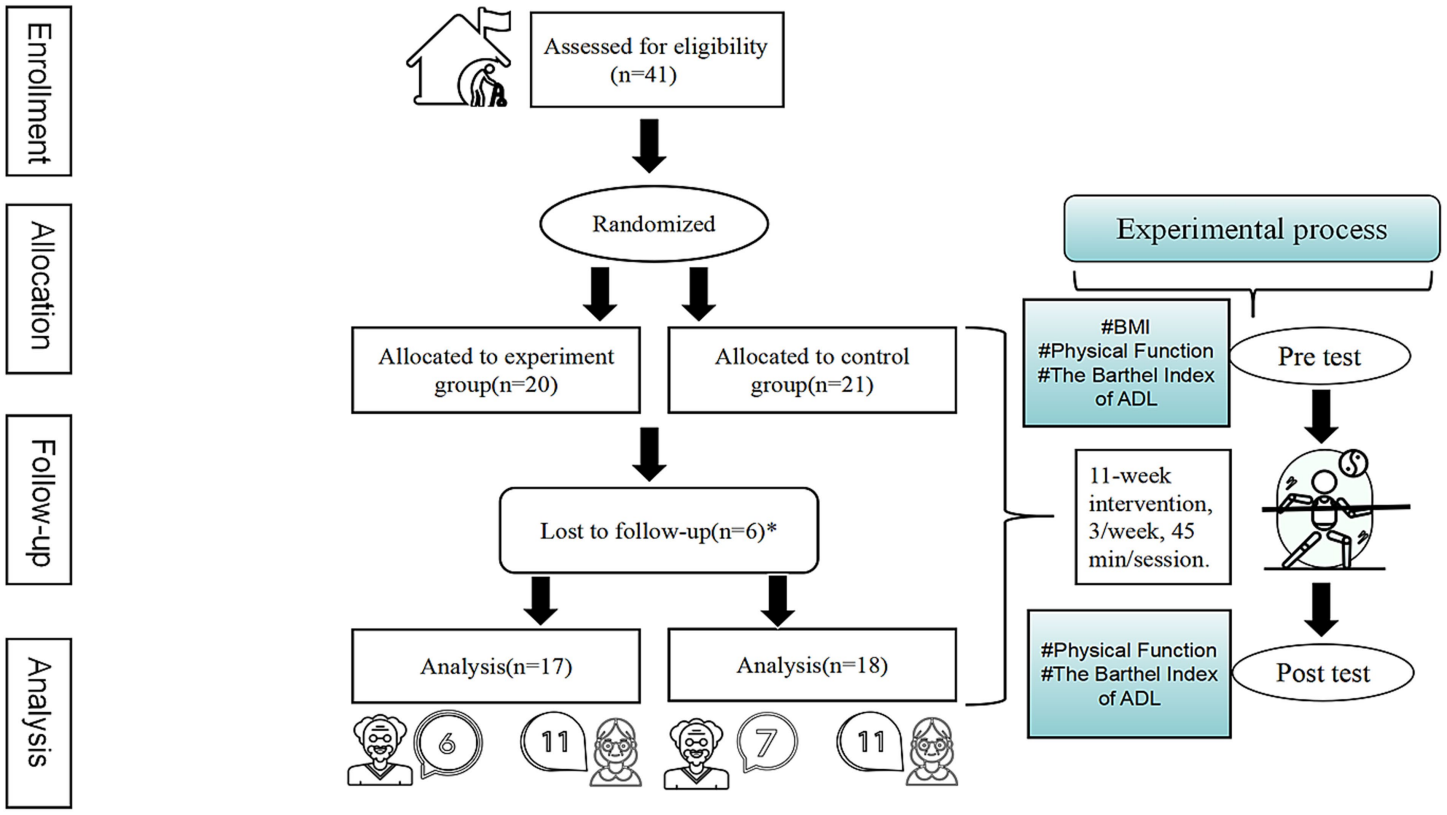
Participant flow chart.

In this study, 41 older adults (15 males and 26 females) were recruited from a senior care center in Nanjing. Due to the relatively small sample size of this study, no additional stratification was performed based on baseline characteristics other than gender. Given that the older adult participants in the study wished to know their group assignments on-site, we were unable to implement blinding during the randomization process. To prevent an imbalance in gender distribution that could affect the study results and reduce the potential confounding effect of gender, this study adopted a gender-stratified randomization strategy. Prior to randomization, we divided the older adult participants into two groups by gender: the male group (15 participants) and the female group (26 participants). The specific procedure was as follows: Each participant was assigned a unique number, the male group used cards numbered from 1 to 15, and the female group used cards numbered from 1 to 26. Each participant determined their own number by randomly drawing a card. Subsequently, two identical sealed opaque envelopes were prepared by a senior care center staff not involved in the study, each containing a different grouping rule: one stated that “odd numbers go to the experimental group and even numbers go to the control group”; the other specified that “even numbers go to the experimental group and odd numbers go to the control group.” Finally, a Taiji Stick instructor who did not know any of the participants randomly selected one envelope, opened it on-site, and the final group assignment was completed in accordance with the rule contained in the envelope. Ultimately, the experimental group comprised 20 participants (7 males, 13 females), while the control group comprised 21 participants (8 males, 13 females). Throughout the intervention, each group experienced the withdrawal of three participants. The dropout rate of this study was 14.63%, which was lower than the universally acknowledged 20%. This result meets the quality standards of similar intervention studies (26). Consequently, The final analysis comprised data from 35 participants. The experimental group included 17 participants (6 males, 11 females), while the control group had 18 participants (7 males, 11 females) (Figure 1). The experimental group participated in Taiji Stick exercise, while the control group did not receive Taiji Stick intervention and any other interventions, maintained their original lifestyle (e.g., eating, sleeping, reading, listening to music, watching movies, etc.), which was supervised by the dedicated personnel from the senior care center. The compliance rate of the experimental group in this study was 95.72%, and the average number of session attendances was 31.59.

### 2.3 Intervention program

**Figure 2:**
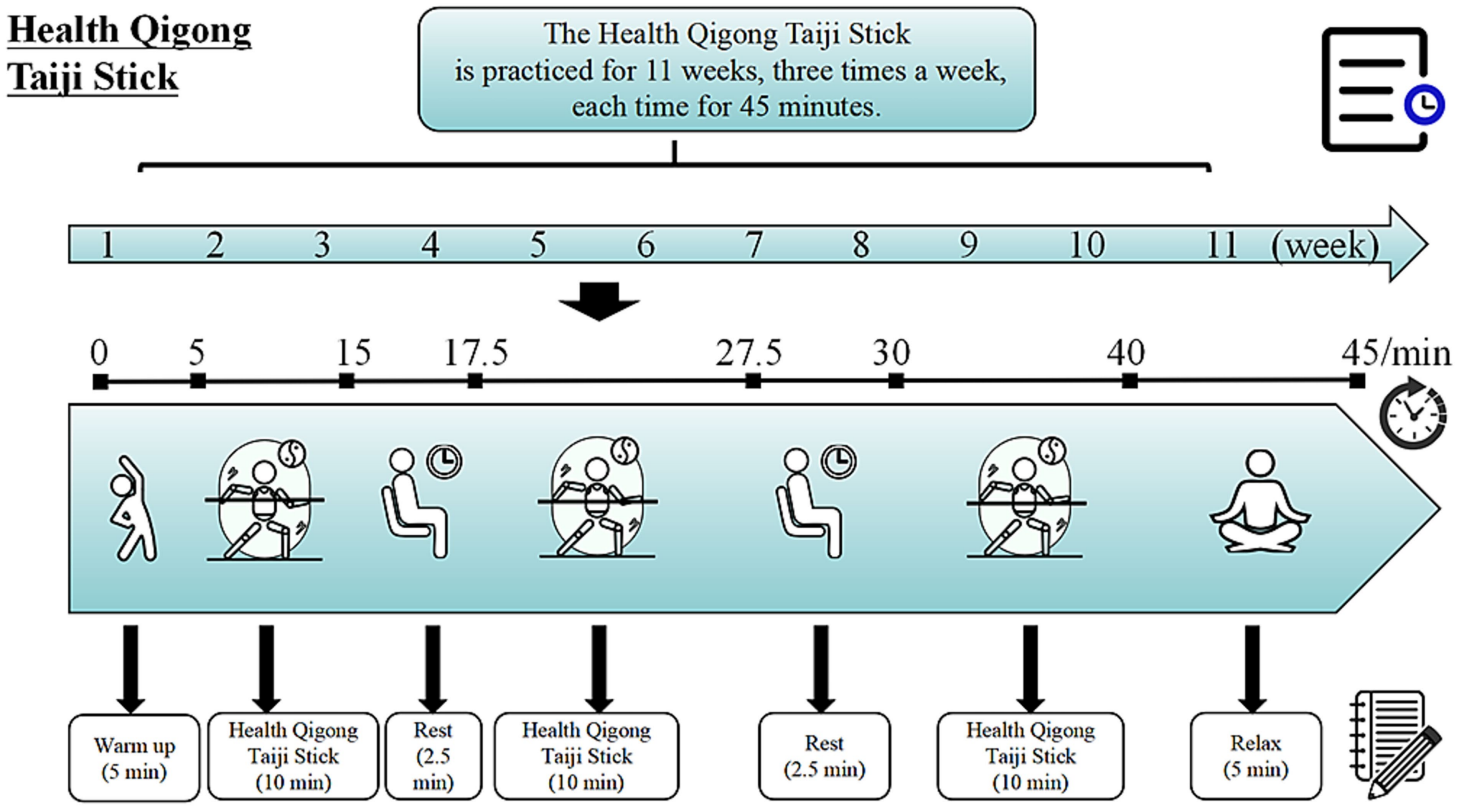
Single intervention procedure.

Taiji Stick exercise (with each wooden stick weighing approximately 0.42 kg on average) was implemented as an intervention program. The intervention group underwent an 11-week exercise regimen, which involved three 45-min sessions per week. Each training session for the intervention group was scheduled from 9:00 to 9:45 in the morning on Monday, Wednesday, and Friday each week. Each session lasted 45 min and was structured as follows: a 5-min warm-up, 10 min of Taiji Stick practice, and a 2.5-min break; followed by another 10-min of Taiji Stick practice and a second 2.5-min break; then a third 10-min of Taiji Stick practice; and finally a 5-min relaxation activity to conclude the session. The instructional content for each session was outlined in Figure 2, with the exercise intensity prescribed at 40% ~ 60% of HRmax, HRmax = 208 – 0.7 × age (27). The weekly and overall instructional plans for Taiji Stick training are detailed in Table 1.

**Table 1:** Health Qigong Taiji Stick exercise program.

Week number	Weekly Training Schedule		
	Monday content	Wednesday content	Friday content
1	Boatman Rows with an Oar (Shao Gong Yao Lu) Left-side movements	Boatman Rows with an Oar (Shao Gong Yao Lu) Right-side movements	Boatman Rows with an Oar Form1(Shao Gong Yao Lu) Complete movements
2	Boat Rows Slowly (Qing Zhou Huan Xing) Left-side movements	Boat Rows Slowly (Qing Zhou Huan Xing) Right-side movements	Boat Rows Slowly Form2(Qing Zhou Huan Xing) Complete movements
3	Wind Kisses Lotus Leaves (Feng Bai He Ye) Left-side movements	Wind Kisses Lotus Leaves (Feng Bai He Ye) Right-side movements	Wind Kisses Lotus Leaves Form3(Feng Bai He Ye) Complete movements
4	Boatman Tows a boat (Chuan Fu Bei Qian) Left-side movements	Boatman Tows a boat (Chuan Fu Bei Qian) Right-side movements	Boatman Tows a boat Form4(Chuan Fu Bei Qian) Complete movements
5	Iron Stick Calms the Sea (Shen Zhen Ding Hai) Left-side movements	Iron Stick Calms the Sea (Shen Zhen Ding Hai) Right-side movements	Iron Stick Calms the Sea Form5(Shen Zhen Ding Hai) Complete movements
6	Golden Dragon Wags Its Tail (Jin Long Jiao Wei) Left-side movements	Golden Dragon Wags Its Tail (Jin Long Jiao Wei) Right-side movements	Golden Dragon Wags Its Tail Form6(Jin Long Jiao Wei) Complete movements
7	Search for Treasure in the Sea (Tan Hai Xun Bao) Left-side movements	Search for Treasure in the Sea (Tan Hai Xun Bao) Right-side movements	Search for Treasure in the Sea Form7(Tan Hai Xun Bao) Complete movements
8	Qi Returns to Dantian (Qi Gui Dan Tian) Ending Stance	Qi Returns to Dantian (Qi Gui Dan Tian) Ending Stance	Qi Returns to Dantian Form8(Qi Gui Dan Tian) Ending Stance
9	Review Form 1	Review Forms 1–2	Review Forms 1–3
10	Review Forms 1–4	Review Forms 1–5	Review Forms 1–6
11	Review Forms 1–7	Review Forms 1–8	Review Forms 1–8 and Ending Stance

### 2.4 Primary outcome measures

#### 2.4.1 Grip strength test

Participants held an electronic grip strength device (ANTA, Electronic Grip Strengthener, Size: 195 × 131 × 33 mm, China), while standing straight with feet positioned about shoulder-width apart and arms hanging loosely by their sides. At the start of the test, each individual was asked to grip the device’s handle with maximum effort using the designated hand. During the test, swinging of the arms or contact with the body was strictly prohibited. Each hand (left and right) was tested twice, and the highest value from the two trials was recorded for analysis.

#### 2.4.2 Five times sit-to-stand test

Subjects sat on a chair without armrests, with a seat height of approximately 43 cm (28). During the test, they were required to maintain an upright posture without leaning against the backrest, the arms were folded across the chest, the eyes facing forward, and the feet placed naturally apart. Without using their arms for support, Five consecutive stand-sit repetitions were performed by each participant as fast as possible. Full knee extension was required upon standing, and participants must return fully to the seated position each time. Each set of five sit-to-stand movements constituted one trial. Three trials were performed in total, with a one-minute rest interval between each of the two trials. The mean time across the three trials was utilized to determine the final result. The Five Times Sit-to-Stand test (5STS) is widely recognized as a valid and credible evaluation of lower extremity strength in older adult individuals (29–31).

#### 2.4.3 Timed up and go test

A chair about 46 cm in height and equipped with armrests was used for participant seating (32, 33), participant’s backs against the backrest and their hands placed on the armrests. Upon hearing the verbal cue“start,” they received instruction to rise from the chair and walk at a comfortable and natural stride to a marker positioned 3 meters in front of them, turn around at the marker, return to the chair by walking, and sit down again with their back against the backrest and their hands on the armrests. Timing began at the verbal cue and concluded once the participant returned to a seated position. The test was performed three times, with a one-minute rest interval between each trial, and the average time of the three trials was recorded as the final outcome. The Timed Up and Go test (TUG) is regarded as a credible and effective approach for assessing dynamic balance in older adults (34–36).

#### 2.4.4 Activities of daily living assessment

The Barthel Index of Activities of Daily Living comprises 10 items. Bathing and Grooming are scored as 0 or 5 points. Dressing, Eating, Toileting, Bowel control, Bladder control, and Stair climbing are scored as 0, 5, or 10 points. Bed-to-chair transfer and The walking score ranges from 0 to 15, in increments of 5 points. The total score varies between 0 and 100, where higher scores represent greater independence and less need for assistance. The Barthel Index of Activities of Daily Living showed an internal consistency with a Cronbach’s alpha coefficient of 0.965 (37, 38).

The specific assessment time points for the above outcomes were as follows: the pre-test was conducted on February 16, 2025, and the post-test was on May 3, 2025. Due to the small sample size, all outcome indicators were completed on the day of testing. No adverse events such as falls or injuries occurred during the intervention period in this study.

### 2.5 Statistical analysis

Statistical analyses were conducted using SPSS 27.0. The normality of the continuous variables was assessed prior to further analysis. The continuous variables were expressed as mean ± standard deviation (Mean ± SD). For baseline data that followed a normal distribution, the independent samples *t*-test was used. For baseline data that did not follow a normal distribution, the non-parametric Mann–Whitney U test was applied. The chi-square test was used to compare categorical variables such as gender between the experimental and control groups. These analyses were conducted to assess the homogeneity of baseline characteristics between the two groups.

A repeated measures analysis of variance (ANOVA) was used to evaluate the within-group and between-group differences in various indicators before and after the exercise intervention in the experimental and control groups. If the time × group interaction effect was not significant, main effects were analyzed. If the interaction effect was significant, simple effects analyses were performed. *Post hoc* multiple pairwise comparisons were performed using the Bonferroni correction. If the sphericity assumption was violated, the Greenhouse–Geisser correction was applied. When the data did not meet the normality assumption, the non-parametric Wilcoxon test was used. Spearman correlation analysis was conducted to explore the relationships among grip strength, lower limb strength, dynamic balance, and Activities of daily living (ADL) in older adult participants following the exercise intervention. Statistical significance was determined using a threshold of *p* < 0.05.

## 3 Results

### 3.1 Baseline homogeneity test

Prior to the exercise intervention, baseline homogeneity between the experimental and control groups was assessed in terms of gender (male/female), age (years), BMI (kg/m^2^), left grip strength (kg), right grip strength (kg), 5STS (second), TUG (second), and ADL (score). Across all examined variables, no statistically significant distinctions were detected between the two groups, indicating that the baseline characteristics were well-matched (Table 2).

**Table 2:** Baseline characteristics (mean ± SD)/median (Q1, Q3).

Variables	Intervention group	Control group	χ^2^	*T*/*Z*	*P*
*N*	17	18	–	–	–
Gender (male/female)	6/11	7/11	0.048	–	0.826
Age (years)	83.71 ± 2.89	85.22 ± 2.78	–	−1.583	0.123
BMI (kg/m^2^)	23.20 ± 2.99	24.18 ± 3.31	–	−0.922	0.363
LGS (kg)	17.41 ± 5.71	17.52 ± 6.10	–	−0.058	0.954
RGS (kg)	19.00 (15.65, 24.45)	17.15 (14.18, 23.68)	–	−0.858	0.391
5STS (second)	15.00 (13.40, 20.00)	16.40 (14.75, 20.85)	–	−1.238	0.216
TUG (second)	15.13 ± 4.04	17.16 ± 3.92	–	−1.511	0.140
ADL (score)	95.00 (90.00, 100.00)	95.00 (88.75, 100.00)	–	−0.052	0.959

LGS, Left grip strength; RGS, Right grip strength; 5STS, Five times sit-to-stand test; TUG, Timed Up and Go test; ADL, Activities of Daily Living.

### 3.2 Changes in physical function and ADL following exercise intervention

#### 3.2.1 Left grip strength

The results of the repeated measures ANOVA demonstrated no significant main effect of time [*F*_(1, 33)_ = 0.703, *p* = 0.408, η_p_^2^ = 0.021, 95%CI (−0.244, 0.587)], no significant main effect of group [*F*_(1, 33)_ = 0.022, *p* = 0.883, η_p_^2^ = 0.001, 95%CI (−3.682, 4.263)], and no significant time × group interaction effect [*F*_(1, 33)_ = 3.961, *p* = 0.055, η_p_^2^ = 0.107]. These findings indicate that Taiji Stick exercise did not significantly improve left grip strength (LGS) in older adult participants (Table 3). Notably, while these results indicate no statistically significant effects, percentage change analyses provide descriptive insights into potential trends. After the intervention, the experimental group showed a 1.3% percentage change in left-hand grip strength, indicating a trend of improvement; the control group exhibited a −3.3% percentage change, indicating a trend of decline.

**Table 3:** The comparisons of outcome measures before and after the intervention (mean ± SD)/median (Q1, Q3).

Variables	Intervention group (*n* = 17)		Control group (*n* = 18)	
	Pretest	Posttest	Pretest	Posttest
LGS (kg)	17.41 ± 5.71	17.64 ± 5.13	17.52 ± 6.10	16.94 ± 6.18
RGS (kg)	19.00 (15.65, 24.45)	19.40 (16.15, 25.20)	17.15 (14.18, 23.68)	16.75 (13.03, 23.48)^b^
5STS (second)	15.00 (13.40, 20.00)	12.30 (9.55, 15.20)^bd^	16.40 (14.75, 20.85)	17.50 (15.50, 23.25)^b^
TUG (second)	15.13 ± 4.04	11.77 ± 3.42^bd^	17.16 ± 3.92	17.92 ± 4.70
ADL (score)	95.00 (90.00, 100.00)	95.00 (92.50, 100.00)^ad^	95.00 (88.75, 100.00)	90.00 (80.00, 95.00)^b^

For comparisons between pre-test and post-test within the same group: ^a^*P* < 0.05, ^b^*P* < 0.01.

For inter-group comparisons at the same test time point: ^c^*P* < 0.05, ^d^*P* < 0.01.

#### 3.2.2 Right grip strength

According to the Wilcoxon signed-rank test, right grip strength (RGS) in the experimental group increased slightly after the intervention compared to the pre-test, this change did not reach statistical significance (*Z* = −1.327, *p* = 0.185, |*r*| ≈ 0.32). However, after the intervention, the percentage change in right-hand grip strength of the experimental group was 2.6%, indicating a trend of improvement. Unlike the experimental group, the control group showed a significant decrease at RGS between the pre-test and post-test assessments (*Z* = −3.338, *p* = 0.001, |*r*| ≈ 0.79). These findings suggest that Taiji Stick exercise may maintain RGS in older adults (Table 3).

#### 3.2.3 Five times sit-to-stand test

Following the intervention, the Wilcoxon signed-rank test, revealed a notable decrease in 5STS completion times within the experimental group compared to baseline measurements (*Z* = −3.551, *p* < 0.001, |*r*| ≈ 0.86). By contrast, the control group experienced a significant rise in 5STS completion times post-intervention (*Z* = −3.529, *p* < 0.001, |*r*| ≈ 0.88). Furthermore, The post-test 5STS results revealed a significant difference between the two groups, as determined by the Mann–Whitney U test [*Z* = −3.433, *p* = 0.001, |*r*| ≈ 0.58, 95%CI (−8.800, −2.800)]. Given that shorter 5STS completion times reflect greater lower limb strength, this study’s findings strongly corroborate that older adults can achieve substantial improvements in lower extremity strength by regularly engaging in Taiji Stick exercise (Table 3).

#### 3.2.4 Timed up and go test

Repeated measures ANOVA demonstrated significant main effect of time [*F*_(1, 33)_ = 16.454, *p* < 0.001, η_p_^2^ = 0.333, 95%CI (0.647, 1.950)], group [*F*_(1, 33)_ = 9.422, *p* = 0.004, η_p_^2^ = 0.222, 95%CI (−6.804, −1.380)], and a notable time × group interaction effect [*F*_(1, 33)_ = 41.388, *p* < 0.001, η_p_^2^ = 0.556]. Analysis of simple effect revealed an absence of marked group differences at baseline TUG completion times [*F*_(1, 33)_ = 2.282, *p* = 0.140, η_p_^2^ = 0.065, 95%CI (−4.768, 0.705)], whereas a significant between-group difference emerged in post-test TUG completion times [*F*_(1, 33)_ = 19.410, *p* < 0.001, η_p_^2^ = 0.370, 95%CI (−8.992, −3.311)]. Within-group comparisons revealed no significant change in TUG completion times in the control group [*F*_(1, 33)_ = 2.908, *p* = 0.098, η_p_^2^ = 0.081, 95%CI (−1.669, 0.147)]. However, the percentage change in TUG completion times of the control group was 4.4%, indicating a negative trend in dynamic balance ability. In contrast, the experimental group exhibited a meaningful reduction in TUG completion times [*F*_(1, 33)_ = 53.489, *p* < 0.001, η_p_^2^ = 0.618, 95%CI (2.424, 4.293)]. As shorter TUG completion times reflect greater dynamic balance, these results suggest that the older adults can achieve significant improvements in dynamic balance ability through Taiji Stick exercise (Table 3).

#### 3.2.5 Activities of daily living

A significant improvement in ADL scores was observed in the experimental group after the intervention, as indicated by the Wilcoxon signed-rank test when compared with the pre-test (*Z* = −2.236, *p* = 0.025, |*r*| ≈ 1.00), while the control group experienced a notable reduction in ADL scores between the pre- and post-test assessments (*Z* = −3.220, *p* = 0.001, |*r*| ≈ 0.89). Furthermore, post-test ADL scores differed significantly between the two groups, as determined by the Mann–Whitney U test [*Z* = −2.725, *p* = 0.006, |*r*| ≈ 0.46, 95%CI (0.001, 15.000)]. Since higher ADL scores indicate greater functional independence, these results suggest that Taiji Stick exercise can significantly improve ADL in older adults (Table 3).

### 3.3 Correlation between physical function and ADL after exercise intervention (the secondary outcome)

**Figure 3:**
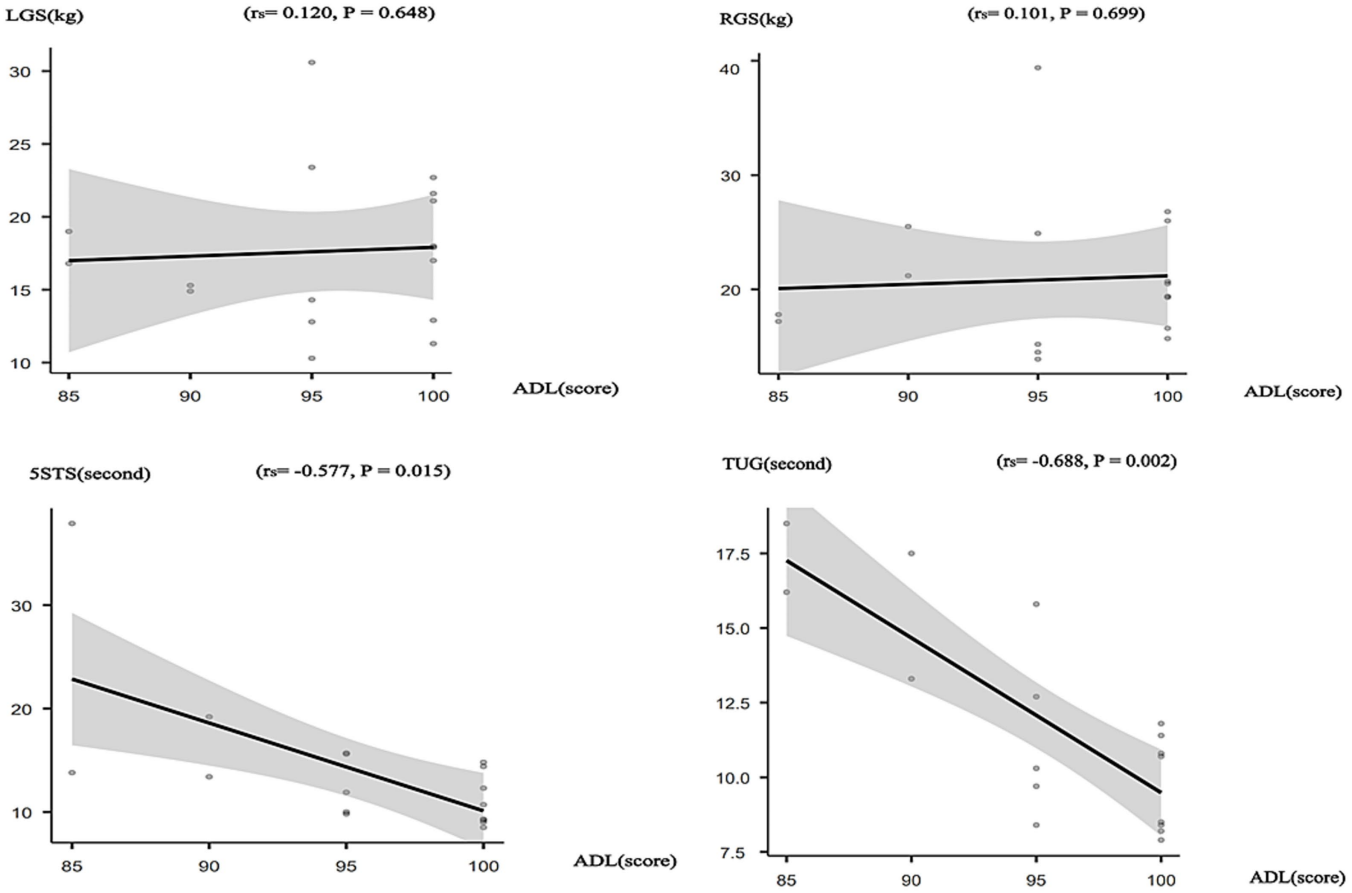
Correlation between Physical function and ADL in the intervention group.

Spearman correlation analysis in the experimental group indicated a positive but nonsignificant correlation between LGS (kg) and ADL scores (*r_s_* = 0.120, *p* = 0.648). Similarly, RGS (kg) showed a positive yet nonsignificant correlation with ADL scores (*r_s_* = 0.101, *p* = 0.699). In contrast, the 5STS completion times (seconds) were significantly negatively correlated with ADL scores (*r_s_* = −0.577, *p* = 0.015). Likewise, there was a marked negative relationship between ADL scores and the times taken to complete the TUG (*r_s_* = −0.688, *p* = 0.002) (Figure 3).

**Figure 4:**
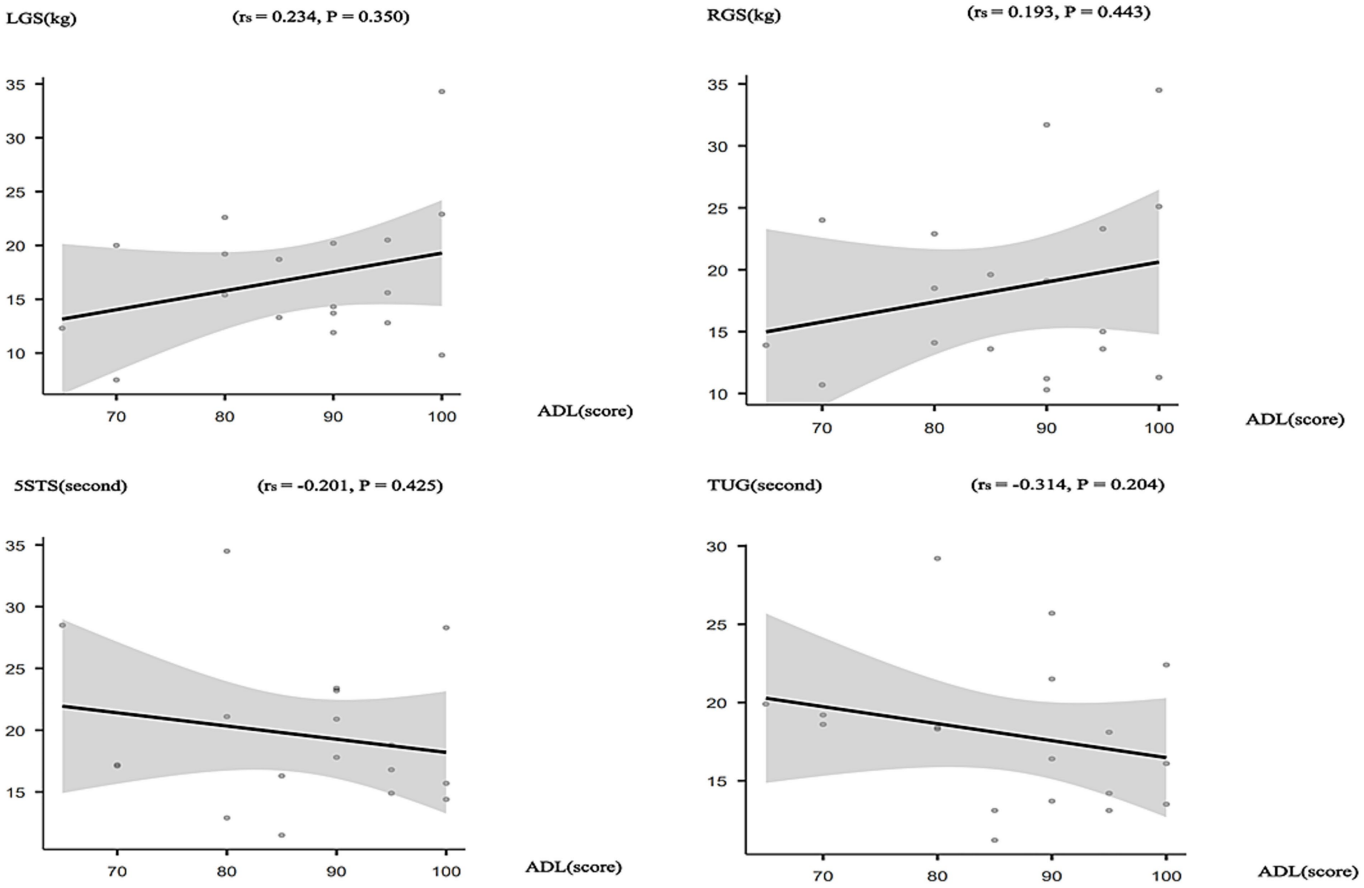
Correlation between Physical function and ADL in the control group.

Spearman correlation analysis in the control group showed a positive but nonsignificant correlation between LGS (kg) and ADL scores (*r_s_* = 0.234, *p* = 0.350). Likewise, RGS (kg) exhibited a positive yet nonsignificant correlation with ADL scores (*r_s_* = 0.193, *p* = 0.443). The 5STS completion times (seconds) were negatively but nonsignificantly correlated with ADL scores (*r_s_* = −0.201, *p* = 0.425). Similarly, the TUG completion times (seconds) demonstrated a negative but nonsignificant correlation with ADL scores (*r_s_* = −0.314, *p* = 0.204) (Figure 4).

The results of the above correlation analysis indicate that improvements in lower limb strength and dynamic balance may contribute to the enhancement of older adult individuals’ ADL.

## 4 Discussion

This study found that an 11-week Taiji Stick exercise intervention significantly improved functional performance and the ability to perform everyday tasks in the older adult, thereby confirming the research hypothesis.

After completing an 11-week Taiji Stick exercise intervention, the grip strength of both the left and right hands of the older adult participants in the experimental group increased, but these increases did not reach statistical significance. Nevertheless, earlier investigations have demonstrated that Taijiquan and Qigong exercises can significantly enhance grip strength. For example, older adults who participated in Yang-style Tai Chi sessions twice weekly for 45 min over 16 weeks demonstrated significant improvements in grip strength (39). Another study reported significant increases in grip strength among older adults following an 8-week Tai Chi (Bafa Wubu) program conducted three times per week for 40 min each session (40). Additionally, older adult women engaging in Qigong exercises twice weekly for 50 min over 8 weeks also exhibited significant enhancements in grip strength (41). However, A meta-analysis indicated that older adult individuals with sarcopenia or frailty showed no significant improvement in grip strength after practicing Tai Chi 2–7 times per week, with each session lasting 30–90 min, for a total duration of 8–48 weeks (42). Meanwhile, another study also found that although there was a tendency for grip strength to increase in frail older adult people after practicing Qigong twice a week, with each session lasting 60 min, for a total of 12 weeks, the improvement did not reach a significant level (43). The grip strength findings in this study are consistent with those obtained from interventions involving Tai Chi and Qigong among older adult individuals with sarcopenia or frailty. Relevant studies have shown that approximately 50% of older adult people over 75 years old suffer from sarcopenia, and about 40% of Chinese older adult people over 60 years old have frailty syndrome (44, 45). Based on the above research results and the average age of the older adult in the experimental group of this study (83.71 ± 2.89), this study infers that the reason why the grip strength of the older adult in the experimental group did not improve significantly after the intervention of Taiji Stick exercise may be due to the presence of sarcopenia or frailty syndrome among the older adult in the experimental group. Subsequent studies will assess whether the participants suffer from sarcopenia and frailty syndrome.

After 11 weeks of Taiji Stick exercise intervention, the 5STS completion time among the older adult in the experimental group was significantly reduced. This result aligns with earlier intervention researches focusing on Tai Chi or Baduanjin. For example, a study found that community-dwelling older women who participated in Tai Chi training twice a week for 60 min per session across 12 weeks exhibited a marked drop in the 5STS completion time (46). Another study reported that older adults with chronic stroke showed a similar improvement after engaging in Baduanjin exercise three times per week, 50 min per session, for 8 weeks (47). A shorter 5STS completion time indicates greater lower limb muscle strength. The significant reduction in the 5STS completion time may be associated with the repeated transitions among various stances commonly performed in Tai Chi or Qigong exercise, such as the bow stance, horse stance, empty stance, and resting stance. These movements are thought to enhanced lower limb muscle strength by facilitating muscle activation, balance control, and weight shifting.

Following the intervention, the TUG completion time among older adults was significantly reduced in the experimental group, aligning with previous findings from earlier researches involving Taijiquan, Baduanjin, Wuqinxi exercises. For instance, seniors experiencing cognitive impairment who participated in Tai Chi training twice weekly, each lasting 60 min over a 14-week period, exhibited a significant reduction in the TUG completion time (48). Another study demonstrated that older adults with chronic stroke who engaged in Baduanjin exercise three times weekly, each session lasted 50 min and was held over 8 weeks, showed a significant decrease in the TUG completion time (47). Furthermore, older adults with knee osteoarthritis who undertook Wuqinxi exercise four times weekly, 60 min per session for 12 weeks, also experienced a notable drop in the completion time for TUG (49). The significant reduction in the TUG completion time may be attributed to the frequent stance transitions commonly practiced in Tai Chi and Health Qigong. These dynamic stance transitions are considered to enhance lower limb muscle strength and improve gait stability. Additionally, movements involving head rotation, flexion and extension of the cervical spine, shoulder rotation, waist twisting and sinking, as well as the joint movements of flexing and extending in the knee, ankle, elbow, along with stretching of both upper and lower limbs, may contribute to improved flexibility, postural control, and balance.

Multiple meta-analyses have confirmed that practicing Tai Chi, Baduanjin, and Qigong can effectively improve lower limb strength (50, 51) and dynamic balance ability (42, 52–54) in the older adult; this study further found that practicing Taiji Stick can also significantly enhance these two aspects. These conclusions provide a clear basis for formulating exercise intervention strategies for the older adult. Accordingly, it is suggested that the older adult take Tai Chi, Baduanjin, Qigong, and Taiji Stick as important ways of their daily exercise, given that regular practice can enhance lower limb strength and improve dynamic balance ability, thus reducing the risk of falls.

After the intervention, significant enhancements in the ADL were observed among older adults in the experimental group. This outcome corresponds with previous studies involving Taijiquan and Baduanjin interventions. For example, one investigation reported that frail older adult individuals who engaged in Taijiquan exercise three times weekly, 30 min each session, over a 12-week period experienced significant improvements in the ADL (55). Similarly, another study reported that frail older adult residents in nursing homes who practiced Baduanjin four times per week, for 35 to 45 min per session over 14 weeks, also showed significant improvements in the ADL (56). All of the aforementioned studies employed the ADL Scale for assessment, which further supports its reliability as a tool for assessing functional status in senior population.

Correlation findings from the present study demonstrated that, following the exercise intervention, strength in the lower extremities and balance performance observed in the intervention group were significantly correlated with the ADL. These findings highlight the value of Focusing on enhancing muscle power in the lower extremities and balance drill in rehabilitation and exercise programs for older adults. Nonetheless, maintaining upper limb strength should not be neglected, so as to better enhance physical functioning and ADL performance in seniors, thereby reducing their risk of falls.

Based on the findings of this study, its results carry significant implications for both clinical practice and public health, as detailed below.

In terms of clinical implications, three key contributions are notable. First, Reducing the risk of falls. Dynamic balance ability and lower limb strength are core elements in fall prevention. Older adults who engage in taiji Stick exercises exhibit significant improvements in lower limb muscle strength and dynamic balance, which in turn reduces their risk of falling. Second, Delaying aging-related functional degradation. Sarcopenia is an age-related, progressive, systemic syndrome characterized by reduced skeletal muscle mass, decreased muscle strength, and impaired function as its core features (44, 57). Through regular stimulation of muscles (especially core muscles and limb muscles), Taiji Stick exercises may play a positive role in delaying the loss of muscle mass and the progression of muscle strength decline, thereby reducing the risk of sarcopenia in older adults. Third, Enhancing activities of daily living and reducing care dependency. Lower limb strength and dynamic balance form the foundation for older adults to independently perform daily activities (e.g., walking, climbing stairs, using the toilet), while upper limb strength is associated with actions like eating and dressing. The findings of this study indicate that Taiji Stick exercise can simultaneously maintain upper limb strength, enhance lower limb strength, and improve dynamic balance ability, thereby promoting older adults’ self-care capacity in daily life.

From a public health perspective, the implications are equally significant. For one thing, Reducing the incidence of accidental injuries. Falls in older adults are likely to result in severe consequences such as fractures, concussions, and intracranial hemorrhage, which not only cause long-term pain and functional impairment but may even be life-threatening. Older adults practicing Taiji Stick can significantly enhance their lower limb muscle strength and dynamic balance ability, thereby reducing the risk of falls and, in turn, decreasing the occurrence of accidental injuries. A reduction in the incidence of accidental injuries among older adults can directly reduce household medical expenditures, alleviate the family’s financial burden and caregiving pressure, and indirectly improve the quality of family life. For another, Enhancing mental health. As a traditional Chinese sport, Taiji Stick uses the stick as a guide and adheres to the practicing essence of “guiding qi to circulate, following with movements, and unifying form and spirit.” Its movement system, which emphasizes waist-centered whole-body movements (e.g., twisting, turning, flexing, extending) integrated with internal activities (such as breathing, mindfulness, and spirit), enables practitioners to achieve physical and mental relaxation during dynamic exercise, thereby mitigating the impact of negative emotions. Additionally, group practice provides a platform for social interaction among older adults, promoting social participation, strengthening their sense of group belonging, and alleviating loneliness through movement communication and experience sharing. These factors altogether form a dual positive effect of “individual emotional regulation combined with group social support,” ultimately contributing to an overall improvement in older adults’ mental health.

Despite the achievements of this study, there are still some limitations. Firstly, only 35 participants were ultimately included in the analysis, which may affect the sample representativeness, stability of the results, and the generalizability of findings. On the one hand, a small-sample experiment may increase the risk of either false negative or false positive results. Specifically, the insufficient statistical power caused by a small sample size makes it harder to detect truly existing effects (i.e., false negatives), particularly when the effect size is small. Conversely, a small sample size may also raise the probability of false positives, as random fluctuations or sampling biases in limited data might be erroneously interpreted as statistically significant effects, thereby distorting the true relationship between variables. On the other hand, the sample of the present study was drawn from a single institution and a specific subgroup (older adult individuals in urban care centers). A small sample size will further exacerbate such “sampling bias,” potentially limiting the generalizability of the research findings to a broader older adult population—including those in urban communities, rural communities, rural care centers, and so forth.

Secondly, failure to implement blinding for participants and assessors can indeed increase the risk of measurement bias. To reduce measurement bias as much as possible, the present study adopted blinding in a specific assessment procedure. Taking the assessment of participants’ activities of daily living (ADL) using the scale as an example, to reduce measurement bias that might arise from assessors’ subjective judgments (e.g., anticipatory bias toward the effectiveness of the intervention group), the following measures were taken in this study: the assessors were professional caregivers from the older adult care center, and they were unaware of both the purpose of this study and the grouping of participants; the assessment was arranged by the leaders of the older adult care center under the pretext of “conducting a routine survey on the ADL of older adult individuals in the center,” thereby helping to mitigate subjective assessment bias that might arise if assessors were aware of the study purpose or grouping information.

Thirdly, This study only adopted gender-stratified randomization for group allocation, which may be subject to interference from other potential confounding factors. To a certain extent, this might affect the comparability between groups and the stability of the study results. Future studies, on the basis of ensuring an adequate sample size, may consider adopting a multi-factor stratified randomization strategy, incorporating key baseline variables such as age, underlying diseases, and exercise capacity into the stratification framework. This would help reduce confounding bias and thereby more accurately reveal the intervention effect of Taiji Stick exercise on older adults.

Fourthly, the study did not examine the persistence of effects following cessation of the Tai Ji Stick exercise intervention. The initial objective of this study was to verify whether Taiji Stick exercise intervention has an improving effect on the strength, balance ability, and activities of daily living (ADL) among older adults immediately after the intervention (i.e., short-term effects), thereby laying a foundation for subsequent studies. In existing Tai Chi intervention studies, an assessment was conducted immediately after the intervention concluded, and if an additional follow-up was scheduled, it was most commonly set at 3 months post-intervention (58, 59). Based on this, we plan to conduct a follow-up assessment at 3 months post-intervention in the subsequent Taiji Stick intervention study to evaluate the maintenance of the effect of Taiji Stick exercise, and thus presenting its exercise benefits more comprehensively.

Lastly, a limitation of this study was that it did not directly assess fall risk, but only inferred from previous findings that improvements in lower limb strength and dynamic balance could help reduce fall risk in older adults, which may limit the persuasiveness of the conclusions. Based on this research, subsequent Taiji Stick intervention studies will directly evaluate fall risk in older adults to enhance the reliability of research conclusions. Future studies will also conduct large-scale, multi-center randomized controlled trials among older adult participants to evaluate the impact of Taiji Stick exercise versus other Chinese traditional exercises, including Taijiquan, Baduanjin, Wuqinxi, and so on. This will not only help further evaluate which intervention produces superior improvements in physical function but also provide reliable evidence to further confirm the stability and generalizability of the results of this study.

## 5 Conclusion

This study demonstrates that an 11-week Taiji Stick exercise intervention significantly enhance lower limb strength and dynamic balance, help maintain upper limb strength, show potential to reduce fall-related risks, and improve the older adults’ capacity to carry out daily tasks. These findings also offer empirical support for the advantages of Taiji Stick practice in older adult individuals, and a necessary foundation for its future promotion and widespread adoption.

## Data Availability

The original contributions presented in the study are included in the article/supplementary material, further inquiries can be directed to the corresponding author.

## References

[1] Koldaş ZL. What is aging and cardiovascular aging? Turk Kardiyol Dern Ars. (2017) 45:1–4. doi: 10.5543/tkda.2017.40350, PMID: 28976370

[2] Naseer F Addas A Tahir M Khan MN Sattar N. Integrating generative adversarial networks with IoT for adaptive AI-powered personalized elderly care in smart homes. Front Artif Intell. (2025) 8:1520592. doi: 10.3389/frai.2025.1520592, PMID: 40017485 PMC11865026

[3] Centers for Disease Control and Prevention (CDC). Trends in aging---United States and worldwide. MMWR Morb Mortal Wkly Rep. (2003) 52:101–6. 12645839

[4] National Bureau of Statistics of China. Demographics. (2025). Available online at: https://www.stats.gov.cn/xxgk/sjfb/zxfb2020/202501/t20250117_1958332.html (Accessed April 5, 2025).

[5] Lin J Ning S Lyu S Gao H Shao X Tan Z. The effects of different types of tai chi exercises on preventing falls in older adults: a systematic review and network meta-analysis. Aging Clin Exp Res. (2024) 36:65. doi: 10.1007/s40520-023-02674-7, PMID: 38472538 PMC10933200

[6] Deandrea S Lucenteforte E Bravi F Foschi R La Vecchia C Negri E. Risk factors for falls in community-dwelling older people: a systematic review and meta-analysis. Epidemiology. (2010) 21:658–68. doi: 10.1097/EDE.0b013e3181e89905, PMID: 20585256

[7] Canning CG Sherrington C Lord SR Close JCT Heritier S Heller GZ. Exercise for falls prevention in Parkinson disease: a randomized controlled trial. Neurology. (2015) 84:304–12. doi: 10.1212/WNL.0000000000001155, PMID: 25552576 PMC4335992

[8] Peeters G van Schoor NM Lips P. Fall risk: the clinical relevance of falls and how to integrate fall risk with fracture risk. Best Pract Res Clin Rheumatol. (2009) 23:797–804. doi: 10.1016/j.berh.2009.09.004, PMID: 19945691

[9] Purdie N. Tai chi to prevent falls in older adults. Br J Community Nurs. (2019) 24:550–2. doi: 10.12968/bjcn.2019.24.11.550, PMID: 31674227

[10] Gerards M Marcellis R Senden R Poeze M Bie R Meijer K. The effect of perturbation-based balance training on balance control and fear of falling in older adults: a single-blind randomised controlled trial. BMC Geriatr. (2023) 23:305. doi: 10.1186/s12877-023-03988-x, PMID: 37198543 PMC10191085

[11] Galloza J Castillo B Micheo W. Benefits of exercise in the older population. Phys Med Rehabil Clin N Am. (2017) 28:659–69. doi: 10.1016/j.pmr.2017.06.001, PMID: 29031333

[12] De Spiegeleer A Beckwée D Bautmans I Petrovic M. Pharmacological interventions to improve muscle mass, muscle strength and physical performance in older people: an umbrella review of systematic reviews and meta-analyses. Drugs Aging. (2018) 35:719–34. doi: 10.1007/s40266-018-0566-y, PMID: 30047068

[13] Gielen E Beckwée D Delaere A De Breucker S Vandewoude M Bautmans I. Nutritional interventions to improve muscle mass, muscle strength, and physical performance in older people: an umbrella review of systematic reviews and meta-analyses. Nutr Rev. (2021) 79:121–47. doi: 10.1093/nutrit/nuaa011, PMID: 32483625

[14] Granacher U Muehlbauer T Bridenbaugh S Bleiker E Wehrle A Kressig RW. Balance training and multi-task performance in seniors. Int J Sports Med. (2010) 31:353–8. doi: 10.1055/s-0030-1248322, PMID: 20180173

[15] Park M Song R Ju K Shin JC Seo J Fan X. Effects of tai chi and Qigong on cognitive and physical functions in older adults: systematic review, meta-analysis, and meta-regression of randomized clinical trials. BMC Geriatr. (2023) 23:352. doi: 10.1186/s12877-023-04070-2, PMID: 37280512 PMC10242998

[16] Gerards MHG McCrum C Mansfield A Meijer K. Perturbation-based balance training for falls reduction among older adults: current evidence and implications for clinical practice. Geriatr Gerontol Int. (2017) 17:2294–303. doi: 10.1111/ggi.13082, PMID: 28621015 PMC5763315

[17] McCrum C Bhatt TS Gerards MHG Karamanidis K Rogers MW Lord SR. Perturbation-based balance training: principles, mechanisms and implementation in clinical practice. Front Sports Act Living. (2022) 4:1015394. doi: 10.3389/fspor.2022.1015394, PMID: 36275443 PMC9583884

[18] Halvarsson A Oddsson L Franzén E Ståhle A. Long-term effects of a progressive and specific balance-training programme with multi-task exercises for older adults with osteoporosis: a randomized controlled study. Clin Rehabil. (2016) 30:1049–59. doi: 10.1177/0269215515605553, PMID: 26396164

[19] Nørgaard JE Andersen S Ryg J Stevenson AJT Andreasen J Oliveira AS. Effect of treadmill perturbation-based balance training on fall rates in community-dwelling older adults: a randomized clinical trial. JAMA Netw Open. (2023) 6:e238422. doi: 10.1001/jamanetworkopen.2023.8422, PMID: 37079305 PMC10119738

[20] Jehu D Paquet N Lajoie Y. Balance and mobility training with or without concurrent cognitive training does not improve posture, but improves reaction time in healthy older adults. Gait Posture. (2017) 52:227–32. doi: 10.1016/j.gaitpost.2016.12.006, PMID: 27939652

[21] Brahms M Heinzel S Rapp M Reisner V Wahmkow G Rimpel J. Cognitive-postural multitasking training in older adults - effects of input-output modality mappings on cognitive performance and postural control. J Cogn. (2021) 4:20. doi: 10.5334/joc.146, PMID: 33748665 PMC7954177

[22] Latham NK Bennett DA Stretton CM Anderson CS. Systematic review of progressive resistance strength training in older adults. J Gerontol A Biol Sci Med Sci. (2004) 59:M48–61. doi: 10.1093/gerona/59.1.m48, PMID: 14718486

[23] Orr R Raymond J Fiatarone Singh M. Efficacy of progressive resistance training on balance performance in older adults: a systematic review of randomized controlled trials. Sports Med. (2008) 38:317–43. doi: 10.2165/00007256-200838040-00004, PMID: 18348591

[24] General Administration of Sport of China. Health qigong. (2020). Available online at: https://www.sport.gov.cn/qgzx/n5402/c957402/content.html (Accessed April 5, 2025).

[25] Baumann H Heuel L Bischoff LL Wollesen B. mHealth interventions to reduce stress in healthcare workers (fitcor): study protocol for a randomized controlled trial. Trials. (2023) 24:163. doi: 10.1186/s13063-023-07182-7, PMID: 36869368 PMC9985281

[26] Cramer H Haller H Dobos G Lauche R. A systematic review and Meta-analysis estimating the expected dropout rates in randomized controlled trials on yoga interventions. Evid Based Complement Alternat Med. (2016) 2016:5859729. doi: 10.1155/2016/5859729, PMID: 27413387 PMC4927989

[27] Tanaka H Monahan KD Seals DR. Age-predicted maximal heart rate revisited. J Am Coll Cardiol. (2001) 37:153–6. doi: 10.1016/s0735-1097(00)01054-8, PMID: 11153730

[28] Weiner DK Long R Hughes MA Chandler J Studenski S. When older adults face the chair-rise challenge: a study of chair height availability and height-modified chair-rise performance in the elderly. J Am Geriatr Soc. (1993) 41:6–10. doi: 10.1111/j.1532-5415.1993.tb05939.x, PMID: 8418126

[29] McCarthy EK Horvat MA Holtsberg PA Wisenbaker JM. Repeated chair stands as a measure of lower limb strength in sexagenarian women. J Gerontol A Biol Sci Med Sci. (2004) 59:1207–12. doi: 10.1093/gerona/59.11.1207, PMID: 15602077

[30] Schaubert KL Bohannon RW. Reliability and validity of three strength measures obtained from community-dwelling elderly persons. J Strength Cond Res. (2005) 19:717–20. doi: 10.1519/R-15954.1, PMID: 16095431

[31] Mong Y Teo TW Ng SS. 5-repetition sit-to-stand test in subjects with chronic stroke: reliability and validity. Arch Phys Med Rehabil. (2010) 91:407–13. doi: 10.1016/j.apmr.2009.10.030, PMID: 20298832

[32] Eekhof JA De Bock GH Schaapveld K Springer MP. Short report: functional mobility assessment at home. Timed up and go test using three different chairs. Can Fam Physician. (2001) 47:1205–7. 11421048 PMC2018519

[33] Hofheinz M Schusterschitz C. Dual task interference in estimating the risk of falls and measuring change: a comparative, psychometric study of four measurements. Clin Rehabil. (2010) 24:831–42. doi: 10.1177/0269215510367993, PMID: 20562166

[34] Ng SS Hui-Chan CW. The timed up & go test: its reliability and association with lower-limb impairments and locomotor capacities in people with chronic stroke. Arch Phys Med Rehabil. (2005) 86:1641–7. doi: 10.1016/j.apmr.2005.01.011, PMID: 16084820

[35] Ries JD Echternach JL Nof L Gagnon Blodgett M. Test-retest reliability and minimal detectable change scores for the timed "up & go" test, the six-minute walk test, and gait speed in people with Alzheimer disease. Phys Ther. (2009) 89:569–79. doi: 10.2522/ptj.20080258, PMID: 19389792

[36] Nightingale CJ Mitchell SN Butterfield SA. Validation of the timed up and go test for assessing balance variables in adults aged 65 and older. J Aging Phys Act. (2019) 27:230–3. doi: 10.1123/japa.2018-0049, PMID: 30117359

[37] Podsiadlo D Richardson S. The timed "up & go": a test of basic functional mobility for frail elderly persons. J Am Geriatr Soc. (1991) 39:142–8. doi: 10.1111/j.1532-5415.1991.tb01616.x, PMID: 1991946

[38] Tornero-Quiñones I Sáez-Padilla J Espina Díaz A Abad Robles MT Sierra RÁ. Functional ability, frailty and risk of falls in the elderly: relations with autonomy in daily living. Int J Environ Res Public Health. (2020) 17:1006. doi: 10.3390/ijerph17031006, PMID: 32033397 PMC7037456

[39] Manson J Rotondi M Jamnik V Ardern C Tamim H. Effect of tai chi on musculoskeletal health-related fitness and self-reported physical health changes in low income, multiple ethnicity mid to older adults. BMC Geriatr. (2013) 13:114. doi: 10.1186/1471-2318-13-114, PMID: 24160867 PMC3870959

[40] Zhang Y Li H Huang R. The effect of tai chi (Bafa Wubu) training and artificial intelligence-based movement-precision feedback on the mental and physical outcomes of elderly. Sensors (Basel). (2024) 24:6485. doi: 10.3390/s24196485, PMID: 39409525 PMC11479303

[41] Yu J Kim J. Effects of a physical activity program using exergame with elderly women. J Korean Acad Nurs. (2015) 45:84–96. doi: 10.4040/jkan.2015.45.1.84, PMID: 25743737

[42] Huang CY Mayer PK Wu MY Liu DH Wu PC Yen HR. The effect of tai chi in elderly individuals with sarcopenia and frailty: a systematic review and meta-analysis of randomized controlled trials. Ageing Res Rev. (2022) 82:101747. doi: 10.1016/j.arr.2022.101747, PMID: 36223875

[43] Tsang HW Lee JL Au DW Wong KK Lai KW. Developing and testing the effectiveness of a novel health qigong for frail elders in Hong Kong: a preliminary study. Evid Based Complement Alternat Med. (2013) 2013:827392. doi: 10.1155/2013/827392, PMID: 24109493 PMC3784263

[44] Vogele D Otto S Sollmann N Haggenmüller B Wolf D Beer M. Sarcopenia - definition, radiological diagnosis, clinical significance. Rofo. (2023) 195:393–405. doi: 10.1055/a-1990-0201, PMID: 36630983

[45] Li S Cui G Yin Y Lv F Yao Y. Association between tea consumption and frailty among Chinese older adults: a cross-sectional study. Front Nutr. (2022) 9:987911. doi: 10.3389/fnut.2022.987911, PMID: 36204378 PMC9531025

[46] Kim CY Je HD Jeong H Jeong JH Kim HD. Effects of tai chi versus Taekkyon on balance, lower-extremity strength, and gait ability in community-dwelling older women: a single-blinded randomized clinical trial. J Back Musculoskelet Rehabil. (2020) 33:41–8. doi: 10.3233/BMR-181493, PMID: 31282402

[47] Yuen M Ouyang HX Miller T Pang MYC. Baduanjin qigong improves balance, leg strength, and mobility in individuals with chronic stroke: a randomized controlled study. Neurorehabil Neural Repair. (2021) 35:444–56. doi: 10.1177/15459683211005020, PMID: 33825587

[48] Li F Harmer P Liu Y Chou LS. Tai Ji Quan and global cognitive function in older adults with cognitive impairment: a pilot study. Arch Gerontol Geriatr. (2014) 58:434–9. doi: 10.1016/j.archger.2013.12.003, PMID: 24398166 PMC3949139

[49] Xiao CM Li JJ Kang Y Zhuang YC. Follow-up of a Wuqinxi exercise at home programme to reduce pain and improve function for knee osteoarthritis in older people: a randomised controlled trial. Age Ageing. (2021) 50:570–5. doi: 10.1093/ageing/afaa179, PMID: 32931545

[50] Liu B Liu ZH Zhu HE Mo JC Cheng DH. Effects of tai chi on lower-limb myodynamia in the elderly people: a meta-analysis. J Tradit Chin Med. (2011) 31:141–6. doi: 10.1016/s0254-6272(11)60029-0, PMID: 21977816

[51] Wang C Liang J Si Y Li Z Lu A. The effectiveness of traditional Chinese medicine-based exercise on physical performance, balance and muscle strength among older adults: a systematic review with meta-analysis. Aging Clin Exp Res. (2022) 34:725–40. doi: 10.1007/s40520-021-01964-2, PMID: 34420189

[52] Cui Z Xiong J Li Z Yang C. Tai chi improves balance performance in healthy older adults: a systematic review and meta-analysis. Front Public Health. (2024) 12:1443168. doi: 10.3389/fpubh.2024.1443168, PMID: 39588165 PMC11586773

[53] Yang Y Li E Gong Z Tualaulelei M Zhao Z Zhang Z. Optimal exercise parameters of Baduanjin for balance in older adults: a systematic review and meta-analysis. Front Public Health. (2025) 13:1541170. doi: 10.3389/fpubh.2025.1541170, PMID: 40177095 PMC11961421

[54] Xie J Guo J Wang B. Comparing the effectiveness of five traditional Chinese exercises in improving balance function in older adults: a systematic review and Bayesian network meta-analysis. PeerJ. (2024) 12:e18512. doi: 10.7717/peerj.18512, PMID: 39553713 PMC11568816

[55] Chen YN. (2022) clinical study of influence of Taijiquan on senile frailty syndrome [master's thesis]. Chengdu: Chengdu University of Traditional Chinese Medicine (2022).

[56] Hou XL. (2018) research of the application of Baduanjin in institutionalized elderly frailty [master's thesis]. Chengdu: Chengdu University of Traditional Chinese Medicine (2018).

[57] Sayer AA Cruz-Jentoft A. Sarcopenia definition, diagnosis and treatment: consensus is growing. Age Ageing. (2022) 51:afac220. doi: 10.1093/ageing/afac220, PMID: 36273495 PMC9588427

[58] Carbonell-Baeza A Romero A Aparicio VA Ortega FB Tercedor P Delgado-Fernández M. Preliminary findings of a 4-month tai chi intervention on tenderness, functional capacity, symptomatology, and quality of life in men with fibromyalgia. Am J Mens Health. (2011) 5:421–9. doi: 10.1177/1557988311400063, PMID: 21406488

[59] Wang C Schmid CH Rones R Kalish R Yinh J Goldenberg DL. A randomized trial of tai chi for fibromyalgia. N Engl J Med. (2010) 363:743–54. doi: 10.1056/NEJMoa0912611, PMID: 20818876 PMC3023168

